# Acting on the call for cervical cancer elimination: Planning tools for low- and middle- income countries to increase the coverage and effectiveness of screening and treatment

**DOI:** 10.1186/s12913-022-08423-2

**Published:** 2022-10-14

**Authors:** Tara Herrick, Kerry A. Thomson, Michelle Shin, Sarah Gannon, Vivien Tsu, Silvia de Sanjosé

**Affiliations:** 1 Market Dynamics, PATH, 2201, Westlake Ave Suite 200, Seattle, WA 98121 USA; 2 Sexual and Reproductive Health Program, PATH, 2201 Westlake Ave, Suite 200, Seattle, WA 98121 USA; 3 Department of Global Health, University of Washington, Seattle, USA

**Keywords:** Cervical cancer screening, Cervical precancer, Cervical cancer treatment, Human papillomavirus (HPV), Planning, Costing

## Abstract

**Introduction:**

Accessible planning tools tailored for low-and middle-income countries can assist decision makers in comparing implementation of different cervical cancer screening approaches and treatment delivery scenarios in settings with high cervical cancer burden.

**Methods:**

The Cervical Precancer Planning Tool (CPPT) was developed by PATH for users to explore and compare the accuracy of screening approaches, what treatment equipment to procure, and how best to deploy treatment equipment in a given country. The CPPT compares four screening approaches: 1) visual inspection with acetic acid (VIA), 2) HPV testing, 3) HPV testing followed by a VIA triage, and 4) HPV testing followed by an enhanced triage test. Accuracy of screening outcomes (e.g., true positives, false positives) is based on published sensitivity and specificity of tests to detect cervical precancerous lesions. The CPPT compares five scenarios for deploying ablative treatment equipment: 1) cervical precancer equipment at every location a woman is screened (single visit approach), 2) equipment only at a hospital level, 3) a single unit of equipment in each district, 4) allowing two districts to share a single unit of equipment, and 5) equipment placed at select district hospitals paired with mobile outreach. Users can customize the CPPT by adjusting pre-populated baseline values and assumptions, including population estimates, screening age range, screening frequency, HPV and HIV prevalence, supply costs, and health facility details.

**Results:**

The CPPT generates data tables and graphs that compare the results of implementing each of the four screening and five treatment scenarios disaggregated by HIV status. Outputs include the number and outcomes of women screened, cost of each screening approach, provider time and cost saved by implementing self-sampling for HPV testing, number of women treated, treatment equipment needed by type, and the financial and economic costs for each equipment deployment scenario.

**Conclusion:**

The CPPT provides practical information and data to compare tradeoffs of patient access and screening accuracy as well as efficient utilization of equipment, skilled personnel, and financial resources. Country decision makers can use outputs from the CPPT to guide the scale-up of cervical cancer screening and treatment while optimizing limited resources.

## Introduction

Cervical cancer is the fourth most common cancer affecting women worldwide and the second most common cause of cancer mortality in women under 50 years of age, resulting in an estimated 342,000 deaths worldwide in 2020 [1]. Nearly 90% of cervical cancer deaths occur in low- and middle-income countries (LMICs) [2], and an estimated 3.7 million women will die from cervical cancer in the next decade unless prevention is substantially scaled-up in high burden LMICs [3]. In recognition of cervical cancer as a significant public health problem, the World Health Organization (WHO) has set ambitious targets to scale-up effective prevention strategies as part of the Global Strategy for cervical cancer elimination, including 90% coverage of HPV vaccination for adolescent girls, 70% of women screened twice in their lifetime with high performance tests by ages 35 and 45, and treatment for 90% of precancerous lesions [4].

Although 116 countries have implemented HPV vaccination for adolescents to some extent since it first became available in 2006 [5], supply, funding, and policy constraints currently limit the reach of this cost-effective strategy in LMICs. To date, 41% of LMICs have introduced HPV vaccination and only 15% of adolescent girls worldwide are fully vaccinated against HPV, most of whom reside in high income countries [6]. Robust screening and treatment programs remain essential for secondary prevention of cervical cancer for the millions of adult women who are not eligible for or will not be reached by HPV vaccination in the coming decades. Recent mathematical modeling from the Cervical Cancer Elimination Modelling Consortium (CCEMC) comparing different combinations of these targets found that although most LMICs could achieve cervical cancer elimination within the next century through 90% HPV vaccination coverage alone, the addition of twice-lifetime HPV testing will accelerate elimination by 11–31 years [7].

With the option to forgo a pelvic exam in favor of self-sampling, the advent of highly sensitive molecular-based DNA testing for HPV holds great promise to increase the effectiveness and reach of cervical cancer screening programs worldwide. The inclusion of a second sequential screening test, commonly referred to as a ‘triage test’, is one option to increase the specificity of a screening approach and identify women at highest risk of developing cervical cancer who require follow-up. WHO guidelines for screening and treatment of cervical precancerous lesions were updated in 2021 [8]. The recommendation for general population women is HPV testing between ages 30 and 49 years with repeat screening every 5–10 years (for a minimum of twice-per-lifetime screening), implemented as either an HPV test-and-treat approach or as an HPV test-triage-treat approach. The recommendation for women living with HIV (WLWH) is an HPV test-triage-treat approach starting at age 25 with repeat screening every 3–5 years, given that WLWH have a higher prevalence of HPV and are more likely to experience persistent infection that progresses to cervical precancer, even after treatment, and at younger ages than women without HIV [9–11].

LMICs face multiple complex decisions and considerations in transitioning from absent or opportunistic screening to adopting the guidelines and ambitious coverage targets from WHO. The predominant approach for cervical cancer screening in LMICs remains naked eye visual inspection with acetic acid (VIA) [12] because this low-cost manual approach can be implemented by clinical staff without specialized laboratory equipment or expertise. New approaches must be considered in the context of important tradeoffs such as the accuracy, costs, training requirements, and infrastructure adaptation needed to scale-up new screening technologies and provide timely linkage to treatment for cervical precancer. For example, implementation of more sensitive screening approaches will identify more screen-positive women who will require follow-up and result in a higher rate of overtreatment, adding increased costs for health systems and burden on women who often need to navigate a complex referral process to access treatment in other facilities.

There is a need for accessible decision-making tools that LMICs can use to guide strategy decisions, clinical guidelines, policy, and operational plans to increase both the coverage of cervical cancer screening as well as program effectiveness in terms of identifying and delivering treatment to women with cervical precancer [13]. In this paper, we introduce the Cervical Precancer Planning Tool (CPPT), an interactive Excel-based tool developed by PATH that allows users to compare outcomes and costs associated with implementing four different screening scenarios and five different treatment approaches.

## Methods

### Overview of the Cervical Precancer Planning Tool (CPPT)

The Cervical Precancer Planning Tool (CPPT) was developed by PATH for users to explore and assess what screening approaches are most appropriate, what treatment equipment to procure, and how best to deploy equipment in a given country. Key outputs are provided in terms of the number of women screened and treated in each scenario and the related costs. Target users of the CPPT include ministry of health (MOH) and other government officials, implementing partners from non-governmental organizations, and funders focused on improving quality and coverage of screening and treatment in LMICs. The CPPT was developed with input from MOHs and advisors from 21 countries across sub-Saharan Africa, Latin America, and Asia. The CPPT was developed using Microsoft Excel (Microsoft, Redmond, WA) to ensure maximum accessibility for target users. The CPPT is accessible in both English and Spanish languages for public download from PATH’s website, along with a training video and fact sheet [14].

The CPPT includes distinct screening and treatment modules that are linked. The country-level, annual, scenario-based tool contains adjustable baseline data for 14 countries in sub-Saharan Africa, Latin America, and Asia that were gathered from literature reviews, global databases (e.g., United Nations Department of Economic and Social Affairs/Population Division, World Population Prospects, UNAIDS AIDSInfo), and primary data collection led by PATH to help define scenarios and vet inputs. The countries that have baseline data inputs in the model are El Salvador, Ethiopia, Ghana, Guatemala, Honduras, Kenya, Malawi, Myanmar, Nicaragua, South Africa, Tanzania, Uganda, Zambia, and Zimbabwe. In addition, any country can use the CPPT by inputting their own data. After the user reviews (or enters) the required baseline data in the screening module, the CPPT automatically calculates results for four different screening approaches. After further selections are made and data are input into the treatment module, the CPPT automatically calculates results for five different treatment approaches. The CPPT defines ‘cervical precancer’ as cervical intraepithelial neoplasia (CIN) grade 2 + . Examples of baseline data are summarized in Table 1. Full details on baseline data, including assumptions and sources, are included in the Screening Input and Treatment Input tabs included in the CPPT.

**Table 1:** Summary of adjustable data inputs used to generate estimates from the Cervical Precancer Panning Tool (CPPT)^a^

Data category	Specific data inputs
District information	Population; health facility count by type
Population demographics	Percentage of population that is female
HIV information	HIV prevalence; percentage of women living with HIV referred for cervical cancer treatment
Cervical cancer screening information	Age range at screening; screening frequency by HIV status; percentage of population to screen; prevalence of CIN2 + and HPV
Cervical cancer screening test information	Sensitivity and specificity of screening tests; costs related to screening
Cervical precancer treatment information	Screen-positive women eligible for ablative treatment; percentage of women treated by scenario
Cervical precancer treatment devices information	Number of existing units by type; percentage of treatment by type (gas or non-gas); maximum number of treatments per device (capacity) per year; costs related to treatment

^a^Full details on baseline data, including assumptions and sources, are included in the Screening Input and Treatment Input tabs that can be viewed once the CPPT has been downloaded by the user

### Estimating the number of women screened and treated

**Fig. 1:**
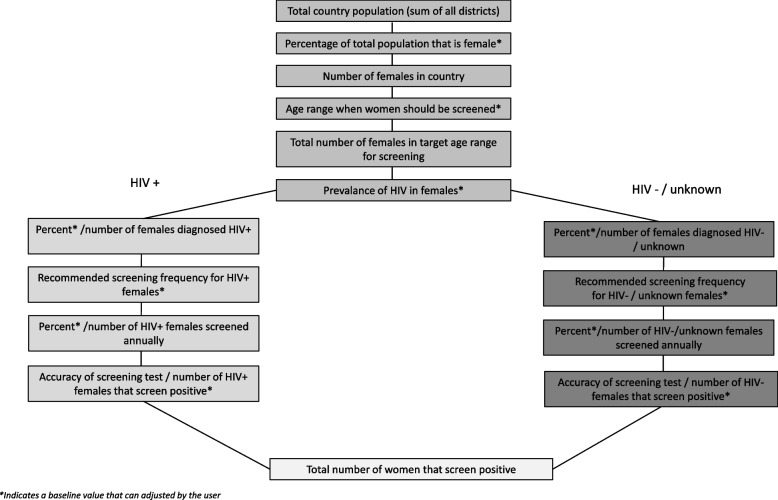
Sequence of estimating the number of women needing screening per year in the Cervical Precancer Planning Tool (CCPT)

The CPPT estimates the number of women screened and treated in each of the scenarios presented, which can be used as one element to calculate tradeoffs between scenarios. The CPPT utilizes population, demographic, and guideline information to calculate the total number of women that need screening in a country and distributes the total number of women screened equally across the number of years that a user inputs for the recommended screening frequency (e.g., every 5 years). The screening results are disaggregated by HIV status, since the prevalence of cervical precancer (CIN2 +) and the recommended screening frequencies are different for those women with and without HIV [8]. Figure 1 presents the sequence used to calculate the number of women who screen positive and need treatment. The outputs are calculated for four potential screening approaches: 1) VIA only, 2) HPV testing only, 3) HPV testing followed by a VIA triage test for HPV positive women, and 4) HPV testing followed by an enhanced triage test for HPV positive women (Table 2). The output for each scenario includes the number of women screened, and the outcomes (based on published sensitivity and specificity for each screening test) for number of true positives, false positives, false negatives, and true negatives. Before moving to the treatment component of the CPPT, the user selects the screening approach of interest for their setting so that the appropriate number of women who screen positive, and thus are eligible for treatment, are subsequently captured in the treatment module.

**Table 2:** Summary of screening approaches included in the Cervical Precancer Planning Tool (CPPT)

Approach	Screening tests	Description
1	VIA alone	A woman in the selected screening age range is screened with a naked eye VIA test. If she screens positive, she is referred to treatment. If a newer approach to visualization becomes available, data specific to this approach can be used in the CPPT instead.
2	HPV alone	A woman in the selected screening age range is screened with an HPV test (physician or self-collected sample, polymerase chain reaction [PCR] or hybrid capture method). If she screens positive for HPV, she is referred to treatment.
3	HPV + VIA triage^a^	A woman in the selected screening age range is screened with an HPV test (physician or self-collected sample, PCR or hybrid capture method). If she screens positive for HPV, she is referred to a VIA triage test. If she screens positive for the triage test (there is a visual suspicion of cervical precancer), she is referred to treatment. Though not factored into this model, women who are HPV + but do not have suspicion of cervical cancer lesions in the triage test should be monitored for persistent infection.
4	HPV + enhanced triage^a^	A woman in the selected screening age range is screened with an HPV test (physician or self-collected sample, PCR or hybrid capture method). If she screens positive for HPV, she is referred to an enhanced triage test. In this Tool, an enhanced triage test refers to a triage test that has an improved performance compared to a traditional triage test (e.g., VIA, colposcopy, or Pap). This improvement should lead to a sensitivity and specificity of at least 80% and/or improvement of 20% over a traditional triage approach. If she screens positive for the enhanced triage test (there is further evidence of cervical precancer), she is referred to treatment. Though not factored into this model, women who are HPV + but do not have further evidence of cervical lesions in the triage test should be monitored for persistent infection.

^a^Scenarios with triage assume that women who are referred to the triage actually complete the test

The CPPT treatment module focuses exclusively on women who are eligible for ablative cervical precancer treatment (cryotherapy or thermal ablation) that can be offered at a primary care level. Of the women who screen positive, 87.7% are estimated as being eligible for ablative treatment [15], and women needing non-ablative treatment (e.g. loop electrosurgical excision procedure [LEEP]) are further excluded from the CPPT. The CPPT contains the number of health facilities, using standard WHO nomenclature to align terminology (i.e., health post, health center, district hospital, provincial hospital, and national hospital) across countries, to estimate the number of equipment units needed for each of five treatment scenarios. These five cervical precancer equipment deployment scenarios examine different strategies for where equipment is placed, including 1) having cervical precancer equipment at every location a women is screened (single visit approach), 2) having equipment at every hospital (hospital treatment), 3) having at least one unit of equipment in each district (district treatment), 4) allowing two districts to share a single unit of equipment if utilization of the equipment is low (district clustering), and 5) having equipment based in select district hospitals and delivered by mobile units to screening sites (hybrid static-mobile) (Table 3).

**Table 3:** Summary of treatment scenarios included in the cervical precancer planning tool (CPPT)

**Scenario**			**Percentage of women receiving ablative treatment**^a^	**Description**^†^
1	Single-visit approach (SVA) for screen and treat		90%	Treatment is available at all health centers and higher-level facilities (excludes health posts). Women receive screening and treatment in one visit. Assumes 10% of women will refuse treatment.
2	Hospital treatment		70%	Treatment is only available at hospitals. If a woman is screened at a health center, she will need to travel to a hospital for a second patient visit to receive treatment. Assumes 30% of women will not go back for a second visit for treatment at a hospital.
3	District treatment		60%	Treatment is only available at select district hospitals. A minimum of one treatment device is placed per district. Additional devices are placed in districts with greater demands. Assumes 40% of women will not travel for a second visit at a hospital in their district for treatment.
4	District clustering		50–60%	Treatment is only available at select district hospitals. Up to two districts with lower demand can share one treatment device. Additional treatment devices are placed in districts with greater demand. If two districts are sharing one treatment device, assumes 50% of women will not travel for a second visit at a hospital in a neighboring district for treatment. If the treatment device is located in the woman’s district, assumes same as Scenario 3.
5	Hybrid static-mobile		80%	Treatment devices are based at select hospitals and treatment is available at these hospitals as well as delivered by mobile units from hospitals to screening sites (health centers or above). Assumes that 20% of women will not go back to a hospital or their local screening site for a second visit for treatment.

^a^These percentages are based on the baseline values for treatment rates, which can be adjusted by the user as necessary in the Treatment Inputs sheet of the CPPT

^†^Terms used in this table are based on WHO classifications of health facilities, but country-specific health facility levels are used when possible in this model

The outputs in the treatment module for each scenario include the total number of women to be treated with ablative procedures, along with the number of units of treatment equipment needed by type and the corresponding equipment utilization rates. The CPPT calculates the number of women indicated for ablative treatment by multiplying the total number of women indicated for treatment (number of true positives plus false positives from the screening module) by the expected treatment completion rate. The CPPT varies treatment completion rates between 50–90% based on the estimated distance a woman would need to travel after screening to obtain the necessary treatment (Table 3). Equipment utilization is calculated by dividing the number of women who are treated per year per unit of equipment by the maximum number of treatments that could occur per year per unit of equipment (based on expert opinion on the health system and device manufacturers input). The CPPT requires users to select a specific type of ablative treatment equipment or mix of multiple types of treatment equipment. The ablative treatment equipment options included in the CPPT are gas cryotherapy, non-gas cryotherapy, and thermal ablation. The logistics of distributing large gas cylinders for cryotherapy in rural areas are challenging in many LMICs. Therefore, the CPPT recommends selecting non-gas approaches for use in rural areas. The adjustable baseline value in the CPPT uses urbanization rates (percent of population living in an urban area) to estimate the proportion of treatment devices that are gas cryotherapy versus non-gas approaches.

### Estimating screening and treatment costs

In addition to estimating the number of women screened and treated in each scenario, the CPPT includes cost as an output for each scenario. In the screening module, the CPPT calculates annual financial costs, including capital equipment and test supplies, for each of the four screening approaches based on the number of units of capital equipment and test supplies needed to screen the women, the price of each test supply, and the expected lifespan of capital equipment. The upfront cost of capital equipment is distributed equally across the lifespan of the equipment. In addition, the CPPT calculates annual economic costs, including health care worker and lab technician costs, based on the provider and lab technician time spent for each patient, their respective annual salaries, and total number of women screened in a country per year. Provider time for screening includes the time it takes to register the woman, prepare for and perform a pelvic examination, collect the sample, and counsel the woman. The lab technician time includes the time it takes to prepare, run, and analyze each specimen. Lastly, the CPPT calculates cost savings and provider time saved related to self-collection for HPV screening approaches, which eliminates the need for a provider to conduct a pelvic exam and collect the sample. Costs to the patient and provider training are excluded from the tool. Baseline costing data in the model, much of which is adjustable by the user, comes from the UNICEF supply catalogue (supplies) [16], the WHO Comprehensive Multi Year Plan (salaries) [17], and peer review studies.

Like the screening module, the treatment module contains health system financial costs and economic costs but excludes patient costs. Capital equipment costs are distributed equally over the five-year lifespan of the equipment, meaning that one-fifth of the equipment costs is captured for the total cost per year. Gas costs are also included as a financial cost if gas cryotherapy is used to treat women. Provider time costs include the time to register the woman, prepare the room, conduct the treatment, and counsel the woman.

## Results

### How to use outputs from the Cervical Precancer Planning Tool (CPPT)

Here, we present select outputs from the CPPT, using Uganda as an example. These outputs can be replicated by downloading the CPPT and selecting ‘Uganda’ on the Screening Inputs tab, using all provided baseline values, and then selecting ‘HPV + VIA triage’ as the screening method on the Treatment Inputs tab.

### Compare screening approaches

**Fig. 2:**
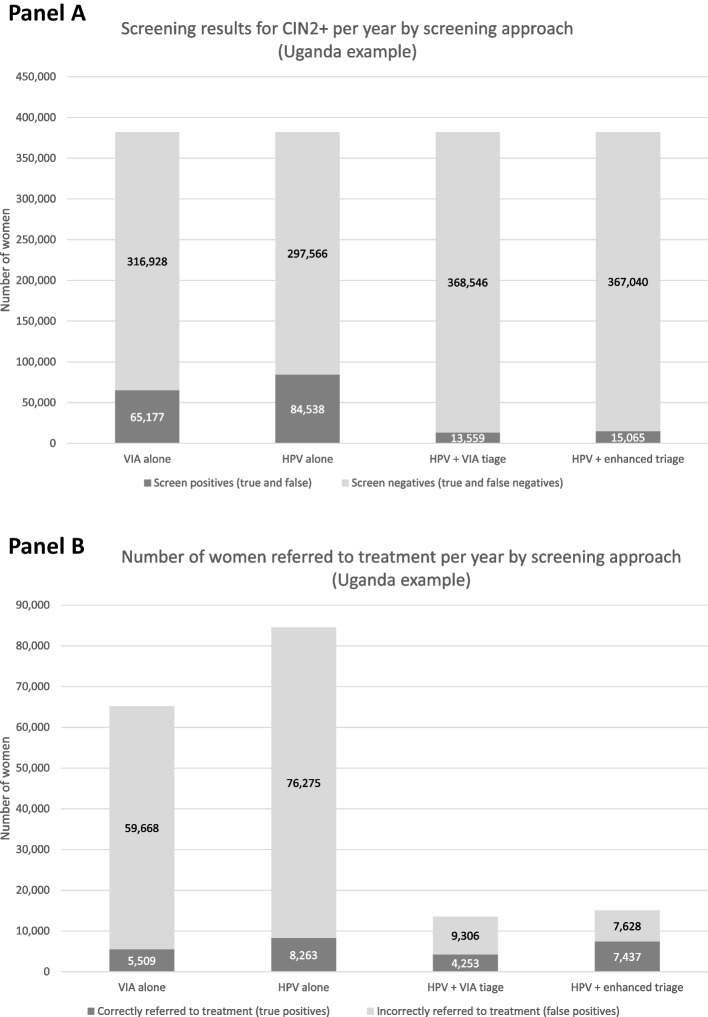
Example screening outputs from the Cervical Precancer Planning Tool (CPPT)

Figure 2 displays example results from CPPT Screening Dashboard for Uganda, where an estimated 382,104 women need cervical cancer screening per year, based on data available at the time of publication [14]. Panel A presents the total number of women who will be correctly referred for treatment (true positives) and incorrectly referred for treatment (false positives) by screening approach. Panel B presents the number of women correctly referred to treatment each year when following each of the four screening approaches included in the CPPT. When ‘HPV testing only’ is used, the largest number of women with CIN2 + are identified, but it also results in a substantial number of women receiving unnecessary treatment given the large number of false positives that are referred for treatment.

Selecting cervical cancer screening and treatment approaches for a given country inherently requires countries to carefully weigh the benefits and consequences of each option and evaluate these tradeoffs within the context of their specific country context, such as cervical cancer burden, geography, budget, and health system capacity. Table 4 summarizes selected tradeoffs and relative comparisons that can be illustrated as a user explores the four screening approaches included in the CPPT, including costs, minimum number of visits required, option for self-sampling, option for same-day treatment, as well as risk of cases missed and risk of overtreatment.

**Table 4:** Relative comparison of tradeoffs across cervical cancer screening approaches for low- and middle-income countries included in the Cervical Precancer Planning Tool (CPPT)

		**Benefits**				**Potential challenges**	
**Screening approach**	**Description and pathway to treatment**	**Screening coverage for a fixed cost**	**Minimum** **clinic visits for screening**	**Option for self-sampling**	**Option for same-day treatment**	**Cases missed**^**a**^	**Overtreatment**
VIA alone	A woman is screened with VIA (regular or enhanced). If positive, she is referred directly to treatment	Lowest	1^b^	No	Yes	High (will vary by provider)	High (will vary by provider)
HPV alone^e^	A woman is screened with an HPV test. If positive, she is referred to treatment without triage	Moderate	0^c^–1	Yes	Only with point of care HPV testing^d^	Lowest	Highest
HPV and VIA triage	A woman is screened with an HPV test. If positive, she is referred to a VIA triage test. If the triage test is positive, she is referred to treatment	Moderate	1^c^–2	Yes	Only with point of care HPV testing^d^	Highest	Low
HPV and enhanced triage^f^	Same as HPV & VIA triage, but the triage test is an enhanced/higher-quality test.^f^	Highest^f^	1^c^–2	Yes		Moderate	Lowest

^a^May be the most important metric if considering screening once in a lifetime

^b^Assuming treatment is available on-site the day screening result is given to woman

^c^Assumes HPV testing could be done with self-sampling

^d^Point of care diagnostic is typically defined as results within 15 min to 2 h (See further for the REASSURED criteria) [18]

^e^Meets WHO definition of “high-precision” [4]

^f^Enhanced triage options currently under evaluation; CPPT tool assumes enhanced triage should lead to a sensitivity and specificity of at least 80%, and/or an improvement of 20% over the baseline triage approach (VIA)

### Compare treatment scenarios

The CPPT estimates 13,559 women will screen positive per year in Uganda when a ‘HPV + VIA’ screening approach is implemented, of which 11,891 women per year will be indicated for ablative treatment. Table 5 presents the number of women who will receive treatment, the number of treatment devices needed, and utilization rates of these treatment devices, depending on the deployment scenario that is used to place treatment devices at different levels of the health system. The highest proportion of women is reached with treatment under Scenario 1, a ‘screen and treat’ approach, which assumes that when treatment units are placed in every location that does screening, 90% of screen-positive women will receive treatment on the same day as receiving their screening test result. However, because only 3.5% of all women screened each year are indicated for treatment (i.e. screen-positive) in the Uganda example, Scenario 1 results in the lowest utilization per device purchased (7 treatments per year). In recognition that distance, transportation, and other opportunity costs affect whether women will complete treatment, the proportion of women treated decreases as treatment devices become more and more distal from a woman’s screening clinic and likely require an additional appointment: a reduction to 70% with hospital placement (Scenario 2), 60% with district placement (Scenario 3) and 50% with district clustering (Scenario 4). Scenario 5, a hybrid static-mobile reaches 80% of women while deploying the least number of treatment devices, resulting in the highest treatment utilization of 832 women per device.

**Table 5:** Summary of treatment outputs calculated in the Cervical Precancer Planning Tool (CPPT) by treatment scenario (Uganda example)

Women treated	Scenario 1	Scenario 2	Scenario 3	Scenario 4	Scenario 5
Number of screen positive women treated per year (total)	10,702	8,324	7,135	6,600	9,513
Number of women treated per year (HIV- or unknown)	8,006	6,227	5,338	4,938	7,117
Number of women treated per year (HIV +)	2,696	2,097	1,797	1,662	2,396
**Treatment devices**					
Total number of devices	1,624	345	112	57	11
Gas cryotherapy^a^	386	82	27	14	3
Non-gas thermal ablation^a^	1,238	263	85	43	9
Equipment utilization (treatments per device per year)	7	24	64	116	832
Equipment utilization^b ^((percentage utilized) per device per year)	1%	2%	6%	11%	80%

Scenarios for deploying treatment devices and (% of eligible women who receive treatment): 1: Screen and treat (90%); 2: Hospital (70%); 3: District (60%); 4: District clustering (50%); 5: Hybrid static-mobile (80%)

^a^Assumes 24% gas cryotherapy and 76% thermal ablation devices based on urbanization data in Uganda

^b^Based on 1,040 treatments per device per year

### Compare costs for different screening approaches and treatment scenarios

**Fig. 3:**
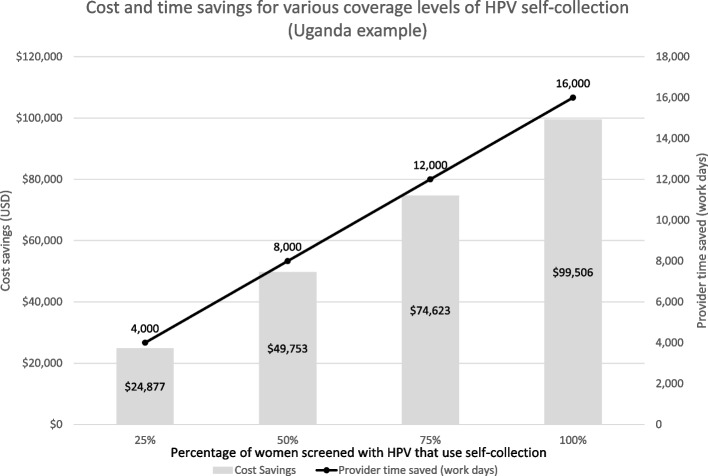
Comparison of cost and time saved by implementing self-collected sampling for HPV testing included in the Cervical Precancer Planning Tool (CCPT)

Table 6 displays costs included in the CPPT Screening Data Tables for Uganda, detailing the total costs in consumables, capital equipment, and staff time, as well as comparison in cost per woman screened and cost per correct diagnosis by each of the four screening approaches included. The total costs calculated by CPPT for the three scenarios that include HPV testing assume that 100% of samples are collected by a healthcare provider. Figure 3 presents savings in costs and provider time that can be achieved if 25–100% of women screened collect their own vaginal samples for HPV testing. In the Uganda example, implementing self-sampling for 75% of women screened with an ‘HPV + VIA’ approach will result in an estimated savings of $74,626 and 12,000 work days of provider time. Table 7 summarizes costs included in the CPPT Treatment Data Tables for Uganda, estimating the total costs in gas and non-gas ablative equipment and provider time for each of the five treatment scenarios to treat the 11,191 women identified as screen-positive and needing treatment when ‘HPV + VIA triage’ is implemented as the screening approach. Development of the CPPT did not include primary data collection or human subjects research and therefore did not undergo review by an ethics committee.

**Table 6:** Summary of costs calculated in the cervical precancer planning tool (CPPT) by screening approach (Uganda example)

**Screening costs (USD)**	**VIA** **only**	**HPV** **only**	**HPV + VIA triage**	**HPV + enhanced triage**^a^
Total cost per year	$145,359	$4,513,234	$4,545,394	$4,704,326
Cost of consumable test supplies	$45,853	$3,908,929	$3,919,073	$4,078,006
Cost of capital equipment (HPV test systems)	$0	$481,912	$481,912	$481,912
Cost of health care provider time	$99,506	$99,506	$121,522	$121,522
Cost of laboratory technician time	$0	$22,886	$22,886	$22,886
Cost per woman screened	$0.38	$11.81	$11.90	$12.31
Cost per correct diagnosis	$0.46	$14.80	$12.36	$12.62

^a^Enhanced triage options currently under evaluation; CPPT tool assumes enhanced triage should lead to a sensitivity and specificity of at least 80%, and/or an improvement of 20% over the baseline triage approach (VIA)

**Table 7:** Summary of costs calculated in the cervical precancer planning tool (CPPT) by treatment scenario (Uganda example)

Screening costs (USD)	Scenario 1	Scenario 2	Scenario 3	Scenario 4	Scenario 5
Total cost per year	$536,065	$126,227	$50,023	$31,517	$23,033
Cost of gas cryotherapy equipment^a^	$142,853	$30,348	$9,852	$5,014	$1,006
Cost of non-gas thermal ablation^a^	$371,373	$78,894	$25,612	$13,035	$2,615
Cost of gas for gas cryotherapy	$16,962	$13,193	$11,308	$10,461	$15,077
Cost of health care provider time	$4,877	$3,793	$3,251	$3,008	$4,335
Cost per woman treated	$50.09	$15.16	$7.01	$4.78	$2.42

Scenarios for deploying treatment devices and (% of eligible women who receive treatment): 1: Screen and treat (90%); 2: Hospital (70%); 3: District (60%); 4: District clustering (50%); 5: Hybrid static-mobile (80%)

^a^Cost assumes 24% gas cryotherapy and 76% thermal ablation devices based on urbanization data in Uganda

## Discussion

Approaches to secondary prevention of cervical cancer are actively evolving, bolstered by the WHO call for elimination, expanding options for HPV assays, emerging alternative technologies for screening, and a resurgence in the use of and normative guidance for thermal ablation. The CPPT is an accessible and user-friendly tool that can aid LMICs in their planning, decision making, and budgeting to expand screening coverage and increase effectiveness to deliver treatment to women with cervical precancer. The four screening approaches and five treatment scenarios were selected based on a combination of current guidelines, common screening and treatment practices in LMICs, and knowledge of emerging technologies for cervical cancer detection and treatment available at the time the CPPT was developed.

### Considerations for selecting cervical cancer screening and treatment options in LMICs

Because VIA does not require specialized equipment or laboratory infrastructure, this approach has the lowest capital cost and is the most straightforward to scale-up in low resource settings. However, the highly subjective nature of the approach results in inconsistent performance across providers and settings, thereby limiting the population-level effectiveness of VIA-based screening programs even when high coverage is achieved in a given country. A cluster-randomized controlled trial of 52 primary health care centers in India comparing both HPV and VIA approaches to no screening intervention found that a single round of HPV-based screening resulted in a 53% reduction in the incidence of advanced cervical cancer and 48% reduction in death after 8 years, whereas no statistically significant benefit was detected in the VIA group [19, 20]. A recent systematic review of 23 studies in LMICs found documented sensitivity and specificity of VIA ranging from 5–100% and 65–98%, respectively [21]. Recognizing that many LMICs still rely on VIA-based screening programs as the primary screening modality, VIA was included in the CPPT as Screening Approach 1. While the CPPT assumes that VIA has 60% sensitivity and 84% specificity for CIN2 + when used as a primary screening test and 51% sensitivity and 88% specificity for CIN2 + when used as a triage test, based off published data, the actual performance in practice will vary across implementation settings. Users of the CPPT can refer to Screening Approach 1 ‘VIA only’ as a baseline against which to compare the implementation of high-precision tests, such as HPV testing, alone or with triage as recommended by normative guidance from both WHO and the International Agency for Research on Cancer [8, 22].

Countries must evaluate and select one or more screening approaches that reflect both their population needs and health system capacity. When implemented as HPV tests using self-sampling, the WHO target of twice-per-lifetime screening requires minimal provider time and could be implemented with innovative outreach approaches outside of fixed health facilities. Based on average HPV prevalence among HIV uninfected women targeted for screening, approximately 12% of women [23] will require provider follow-up to assess treatment needs while HPV negative women can be informed of results via phone or digital health solutions, eliminating the need for women to travel to facilities solely to obtain negative screening results. Although studies from LMICs are limited, results to date have found that HPV testing with self-sampling is more cost-effective than VIA as the standard of care [24, 25]. Mathematical modeling specific to Uganda found that once-per-lifetime HPV-testing as part of a campaign with self-collected sampling was cost-effective as compared with provider-collected samples when ≥ 75% coverage is achieved [26]. In 2020, WHO released a step-by-step guide for introduction and scale-up of HPV testing that program leaders can use for planning, implementation, procurement, monitoring, and scale-up of HPV testing [27].

In recognition of the differences in HPV prevalence, cervical cancer risk, and recommended screening intervals among WLWH, the CPPT disaggregates results in the screening approaches by HIV status. With high sensitivity but low specificity for CIN2 + , the implementation of HPV testing alone among WLWH, for whom HPV prevalence can be 50% or higher [28, 29], can result in overtreatment due to the detection of otherwise transient HPV infections [30]. Countries with a high dual burden of HIV and cervical cancer will need different approaches for these women, such as a highly accurate triage test to further identify those needing treatment or more frequent screening among WLWH. WLWH established in HIV care and antiretroviral therapy programs may be at less risk of loss to follow-up from integrated cervical cancer screening than their HIV uninfected peers who have fewer routine touchpoints with the health system. The current version of the CPPT allows the user to enter only one age range for screening; this cannot be adapted for HIV status to reflect the current WHO recommendation to start screening for WLWH at 25 years vs. 30 years for women without HIV. However, the flexible nature of the CPPT allows a user to view the CPPT separately for women with and without HIV to consider the nuances in deploying different, but parallel, country level screening and treatment strategies based on HIV status.

The fourth screening approach (‘HPV + enhanced triage’) was included to leave a placeholder for novel approaches that are still under development or evaluation, especially triage tests that can further identify HPV positive women with the highest risk of developing cervical precancer and/or cancer.

Extended HPV genotyping, viral and host methylation of specific genes, dual staining for p16/Ki67, and detection of viral oncoproteins E6/E7 are examples of promising approaches to further identify HPV positive women at highest risk of cervical cancer [31, 32]. An additional approach currently under evaluation that may be especially suited for low-cost point-of-care screening in LMIC settings combines advances in digital images captured by a smartphone [33] or other dedicated device with the automated reading and scoring of cervical cancer risk through the application of artificial intelligence algorithms to cervical cancer images [34]. Preliminary data suggests this automated visual evaluation (AVE) approach demonstrates high performance when using high quality images such as cervigram images [35, 36]. These innovative approaches need to be fully validated in clinical settings before wide implementation is recommended.

The overall effectiveness of a country’s efforts on secondary prevention of cervical cancer depends both on accurate identification of women at risk of cervical cancer and then successful linkage to and completion of treatment. The 2019 WHO Guidelines for the Use of Thermal Ablation for Cervical Pre-Cancer Lesions recommend thermal ablation as a treatment alternative to cryotherapy for both HIV infected and HIV uninfected women [37]. In addition to shorter treatment application times, battery-powered thermal ablation devices offer the practical advantage of being more portable and reliably available on demand as compared to treatment devices that require electricity or consistent supply of refrigerant gas. Strategic deployment of thermal ablation has the potential to increase treatment completion for screen-positive women. The CPPT also includes non-gas cryotherapy devices given there are examples of its use in some settings, but to our knowledge, the use of this type of equipment is quite rare. Because it is more difficult to achieve complete visualization of the cervix in older and menopausal women, countries must take into consideration that a lower proportion of older screen-positive women will be eligible for thermal ablation. The evidence comparing cure rates of thermal ablation among women with and without HIV is currently limited, but suggestive that countries need to build in follow-up visits following thermal ablation among WLWH to monitor for recurring lesions [38, 39].

### Comparing the CPPT to other available planning resources

Several resources are available to countries as they embark on revising clinical guidelines, adapting health systems, and planning budgets to improve cervical cancer screening and treatment, including the Cervical Precancer Planning Tool (CPPT) presented here, Improving Data for Decision Making in Global Cervical Cancer Programmes (IDCCP) Toolkit and the Cervical Cancer Prevention and Control Costing (C4P) Tool, and cost-effectiveness analyses. Table 8 compares selected features across these currently available resources.

**Table 8:** Comparison of selected tools and approaches for planning and costing interventions for secondary prevention of cervical cancer

Characteristic	Cervical Precancer Planning Tool (CPPT)	Cervical Cancer Prevention and Control Costing (C4P) of the Improving Data for Decision Making in Global Cervical Cancer Programmes (IDCCP)	Cost-effectiveness analysis
Designer	PATH	World Health Organization (WHO)	Varies, usually academic research organizations
Language	English, Spanish	English, Spanish	Depends on the analyst & country context
Purpose	Planning	Program planning and monitoring, and surveillance	Health technology assessment (e.g. interventions, diagnostics), healthcare policy decision making
Audience(s)	Country decision makers	Country decision makers	Policy decision makers, other modelers, international normative bodies, advisory groups shaping clinical guidelines
Screening and treatment strategies	4 screening approaches and 5 treatment scenarios methods (compared simultaneously)^a^	Options for 4 screening methods, (up to 3 can be compared simultaneously) and 7 treatment methods^b^	Typically, a base-case comparing a new approach to standard of care Fully parameterized, includes sensitivity analyses for robustness
Program and service outputs	Screening, diagnosis, and treatment of cases eligible for ablation	Screening, diagnosis, and treatment services, microplanning, training, social mobilization and communication, supervision, monitoring and evaluation	
Cost outputs	Economic costs (e.g., provider time) Financial costs (e.g., equipment, testing supplies)	Economic costs (e.g., provider time) Financial costs (e.g., equipment, testing supplies)	Incremental Cost-Effectiveness Ratio (cost per life-year gained, quality-adjusted life years, disability-adjusted life years)
Customization	User can choose between pre-programed defaults or customizing data input(s)	User can choose between pre-programed defaults or customizing data input(s)	High level of customization
Unit of analysis	National level	National or regional level	Can be customized, but typically at the national level
Target population and timeframe	Using current country-level population data on women eligible for screening and treatment for 1 year of implementation (static model)	Using current country-level population data on women eligible for screening and treatment for up to 5 years of implementation (static model)	Can range from static, dynamic, to micro-simulation model, with projected target population. Adjustable analytic horizon; typically follows cohort of women for life given slow disease progression
Analytic horizon	Prospective analysis only	Both prospective and retrospective analysis possible	Typically prospective, but retrospective analysis possible
Time until results	A few hours	Months of time for WHO country visit and follow-up analyses	Typically 12 + months to develop and parameterize a full model
Software platform	Microsoft Excel	Microsoft Excel	Various programming languages
Cost to end-user	Free to download	Moderate cost to engage a WHO trained facilitator	Analysis typically grant-funded and results published in peer-reviewed literature (may be open-access)
Level of experience needed by end-user	No prior experience needed. End-user follows detailed instructions and training video provided	End-user relies on WHO facilitator, does not interact with models directly	End-user relies on modeling experts, does not interact with models directly

^a^Screening approaches: VIA alone, HPV testing alone, HPV testing and VIA triage, HPV testing and enhanced triage; Treatment scenarios: Single-visit approach for screen-and-treat, hospital treatment, district treatment, district clustering, hybrid static-mobile with cryotherapy

^b^Allows for combinations methods for the transition period; Screening options: VIA, VILI, cytology, HPV testing; Diagnostic options: Colposcopy, biopsy, histopathology; Treatment options: LEEP, cryotherapy, cold knife conization, thermal ablation, chemotherapy, radiotherapy, surgery

The IDCCP Toolkit was developed by WHO and a consortium of partners with the goal of developing global standards, tools, and guidance for improving the availability and use of high-quality data for decision-making in cervical cancer programs in LMICs. Published in 2018, the IDCCP includes five sections, four of which provide guidance and tools that countries can adapt independently to gather information on data systems and implement new data collection for monitoring and evaluation of cervical cancer prevention programs: 1) Rapid situational assessment of data and data systems, 2) Population-based survey modules, 3) Patient and program monitoring, and 4) Facility-based surveys. The fifth section, “Prevention and control costing: analysis and planning module for screening and treatment”, introduces the country decision makers to the WHO Cervical Cancer Prevention and Control Costing (C4P) Tool [40]. The C4P is an Excel-based data analysis tool, designed specifically to allow health program managers and planners to estimate, analyze and synthesize costs for cervical cancer programs and services. Use of the C4P requires a facilitator; countries engage the services and expertise of a trained WHO facilitator who implements the C4P in tandem with involvement from national government officials and other stakeholders, a multi-month process that can require funding for in-country data collection.

Increasingly, comparative modeling and health economic analyses are conducted to guide decision making, priority setting, and resource allocation for health care systems. Cost-effectiveness analyses (CEA) estimate both the costs and health gains of a given intervention, relative to an alternative intervention, by estimating how much it costs to gain a unit of a health outcome (e.g. cost per life-year gained, quality-adjusted life years, disability-adjusted life years). Therefore, CEA can be a key resource for LMICs where financial resources are limited and the benefits of health interventions and approaches for prevention and treatment need to be maximized, such as adopting new approaches for improving screening and treatment for cervical cancer. Given the level of expertise required, cost-effectiveness analyses are most often conducted by specialized academic teams funded through research grants. These analyses take considerable time and dedicated funding, especially when country-specific data collection is needed to parameterize the model and include sensitivity analyses to evaluate assumptions. There is considerable variability in the rigor of cost-effectiveness studies, although the recently published HPV-FRAME [41] issued a consensus statement and quality-based framework that can be used to guide new and evaluate existing epidemiologic and economic HPV models. The level of resources required can be a barrier to many LMIC governments independently initiating cost-effectiveness analyses of cervical cancer screening and treatment strategies, although efforts are made to generalize findings across LMICs or regions and results are typically disseminated through published literature and synthesized in normative guidelines from WHO.

## Conclusion

Achieving 70% coverage of cervical cancer screening and 90% treatment for screen positive women are ambitious goals set by the WHO, but progress can be achieved when guided by evidence-based decisions and concrete action steps. Advances in normative guidelines and planning tools, such as the CPPT, can aid decision makers and program planners as they develop new cervical cancer control strategies that harness the most effective screening and treatment technologies and implementation approaches for their country’s needs.

## Data Availability

The Cervical Precancer Planning Tool (CPPT), including the outputs and data included here, are publicly available by downloading the CPPT at https://www.path.org/programs/market-dynamics/cervical-precancer-planning-tool/
